# Beliefs About Perioperative Opioid and Alcohol Use among Elective Surgical Patients Who Report Unhealthy Drinking: A Qualitative Study

**DOI:** 10.1093/pm/pnab104

**Published:** 2021-04-23

**Authors:** Anne C Fernandez, Lewei A Lin, Angela R Bazzi, Jeff Boissoneault, Brian Borsari, Frederic Blow

**Affiliations:** 1 Department of Psychiatry, University of Michigan, Ann Arbor, Michigan; 2 Department of Psychiatry, University of Michigan and Center for Clinical Management Research, HSR&D, Veterans Affairs Health Care System, Ann Arbor, Michigan; 3 Herbert Wertheim School of Public Health and Human Longevity Science, University of California, San Diego, California; 4 Department of Clinical and Health Psychology, University of Florida, Gainesville, Florida; 5 Department of Psychiatry and Behavioral Sciences, San Francisco Veterans Affairs Health Care System, University of California San Francisco, San Francisco, California, USA

**Keywords:** Opioid Analgesics, Alcohol Use, Surgery, Pain, Polysubstance Use

## Abstract

**Objectives:**

Elective surgical patients with unhealthy alcohol use have unique pain management needs and addiction risk factors that are relevant to surgical preparation and recovery. This descriptive qualitative study sought to better understand patients' beliefs and behaviors related to opioid use, alcohol use, and pain management in the perioperative context.

**Design:**

We conducted individual semi-structured interviews between July 2017 and March 2018.

**Setting:**

A large Midwestern academic health system.

**Subjects:**

Participants were elective surgical patients meeting unhealthy alcohol use criteria, recruited from the health system’s preoperative anesthesia clinic.

**Method:**

Semistructured interview guides explored beliefs and behaviors relating to alcohol and opioid use, health status, and surgical care. Interview recordings were transcribed and coded for thematic analysis.

**Results:**

Among 20 elective surgical patients (25% female), we identified three key themes regarding alcohol use, opioid use, and their co-use before and after surgery. First, desires and intentions to use opioids for postoperative pain management varied widely, even before opioids were prescribed. Second, some participants described alcohol as a preferred pain management strategy. Third, participants held a range of beliefs about the risks and benefits of alcohol and opioid co-use.

**Conclusions:**

Appropriate assessment of beliefs and intentions regarding opioid and alcohol use could help identify patients most vulnerable to new opioid problems and unhealthy alcohol use in the context of perioperative surgical pain. These findings have important implications for perioperative pain management.

## Introduction

Individuals with unhealthy alcohol use (defined as > 2 drinks/day on average) prior to surgery experience increased risk of postoperative complications [1–3] and may have more complex pain management needs [1, 4]. They can experience alcohol-induced hyperalgesia [5, 6] particularly if they withdraw from alcohol in the context of surgical preparation or recovery [7]. Individuals with unhealthy alcohol use can also experience cross-tolerance to opioid-based pain medications after surgery [8–10]. Furthermore, the increased likelihood of postoperative complications can increase and prolong postoperative pain and use of prescribed opioids. All of these factors highlight the complex pain management needs of surgical patients who also consume alcohol.

Individuals with unhealthy alcohol use are more vulnerable to over-using and misusing opioids commonly prescribed for surgical pain [11–18]. In addition, those with unhealthy alcohol and drug use are more likely to die from an overdose in the 30 days after surgical discharge [19, 20]. Alcohol and opioid co-use increase respiratory depression and risk of mortality beyond what would be observed with the use of either drug class alone [21,22].

Importantly, alcohol itself is a potent analgesic [23], comparable to morphine for acute pain [24, 25]. A blood alcohol concentration of 0.08% induces a moderate to large reduction in pain intensity, with increasing analgesic effects in a dose-dependent fashion [23]. As many as one in four individuals with pain conditions report using alcohol to manage pain [26]. While drinking alcohol reduces pain in the short-term, long-term alcohol use is linked to higher levels of pain and physical impairment [27]. The strong association between unhealthy alcohol use and chronic pain is well-established and attributed to complex biopsychosocial pathways [27, 28]. To date, the possibility that surgical patients may use alcohol to manage acute surgical pain has not been explored.

Unhealthy alcohol use complicates perioperative pain management and opioid prescribing. Nevertheless, very little research has investigated patient’s perceptions regarding co-use of alcohol and opioids to manage pain during the perioperative period. The goal of this descriptive qualitative study was thus to explore beliefs, intentions and behaviors related to opioid and alcohol use and co-use for pain management before and after surgery. An improved understanding of these topics could inform the development of interventions to address patients’ unhealthy alcohol use, opioid misuse, addiction-related risks, and pain management needs following surgery while also generating hypotheses for future research with this vulnerable group.

## Methods

### Study Design and Sample

We conducted a descriptive qualitative study [29–32] involving one-time, semistructured interviews with the primary goal of understanding individuals’ beliefs, intentions, and behaviors related to perioperative alcohol and opioid use and co-use. All data were collected from a large academic medical center located in the mid-western United States. We obtained written informed consent from all participants prior to interviews. The university’s Institutional Review Board approved all study protocols. The data presented here originated from the intervention development phase of a multi-phase study aiming to develop and implement a pre-operative alcohol brief intervention (ASPIRE study: Clinicaltrials.gov Identifier: NCT03929562). This study recruited from the medical center’s preoperative anesthesia clinic in person and by telephone using a convenience sampling approach. Eligibility included (1) age 18 to 75 years old; (2) able to speak English; (3) scheduled to have an elective or semi-elective surgical procedure in the next 90 days; and (4) having a score of ≥ 4 (women) or ≥5 (men) on the Alcohol Use Disorders Identification Test, Consumption Questions (AUDIT-C), as detailed below [33]. Participants were excluded from the larger study if they were scheduled for surgeries using local anesthesia or were having bariatric or transplant surgery, due to the in-depth psychosocial screening and alcohol-related exclusion procedures that usually precede these surgeries. After determining through team discussions that we had reached thematic saturation for qualitative participants with less severe alcohol use, we then used purposive sampling to recruit additional participants with more severe alcohol use (e.g., AUDIT-C ≥ 7) [32, 34]. Of the 383 individuals screened for the larger study, 360 (96.3%) provided complete data, and of those, 66 (18.1%) were eligible and 298 (81.9%) were ineligible. Twenty participants completed qualitative interviews. Surgery cancellation and being too busy prior to surgery were common reasons given for refusing to participate the qualitative interviews.

### Data Collection

From October 2017 to March 2018, two female researchers (a PhD-level, faculty member and a bachelor’s-level study team member) conducted one-time semistructured qualitative interviews. They received qualitative research and interviewing training through qualitative experts and workshops at Brown University and University of Michigan. Contact with participants was limited to research participation. Participants knew that interviewers were study team members and employees of the university. Prior to interviews, interviewers told participants that the goals of the research included “better understand[ing] surgical patients’ stress, health, and alcohol use.” Interviewers used a semi-structured guide organized by key domains including open-ended topic questions and detailed optional probes. The interview guide was reviewed by surgeons, health services researchers, and qualitative experts prior to data collection. Minor iterative changes to guides were made between interviews as new knowledge was obtained. Interviews lasted approximately 75 minutes and took place in office spaces at the university or in libraries, or were completed over the phone (to reduce transportation or scheduling challenges). One interviewer and one participant were present for each interview. The interviewer made field notes during interview. All interviews were audio-recorded for professional transcription. Personal identifiers were not retained in transcripts and they were not returned to participants.

### Quantitative Measures

Demographic characteristics (including age, gender, race/ethnicity) were collected via self-report and from electronic health records. To assess alcohol use, we administered the AUDIT-C, a 3-item screening tool that detects hazardous drinking or the presence of active alcohol use disorders [33]. Scores range from 0 to 12. The AUDIT-C has been found to have good internal consistency among men and women [35]. We used an eligibility cutoff of ≥4 (women) or ≥5 (men). Our cutoffs were chosen based on research indicating a score of ≥ 5 on the AUDIT-C is linked to an increased incidence of postoperative complications in men [36]. A cutoff of 4 was chosen for women to adjust for known gender differences in alcohol-related metabolism and health problems [37]. To assess tobacco use, prescription drug use for non-medical reasons, and illicit drug use, we used items from the Quick Screen of the National Institute on Drug Abuse Modified version of the Alcohol, Smoking, and Substance Involvement Screening Test (NM ASSIST) [38], which is adapted from the World Health Organization ASSIST (version 3.0) [39]. The Quick Screen of the NM ASSIST assesses lifetime substance use using a Likert scale. We modified it to assess past 3-month drug use.

### Data Analysis

Coding began after four transcripts were available and proceeded in an iterative fashion until additional interviews yielded redundant information related to preexisting themes and no additional new themes or codes emerged from the data [40]. When coding, we considered risk and protective theory as an underlying framework, which posits that risk and protective factors directly influence the likelihood of developing a substance use problem [41]. Risk factors include beliefs that may increase the likelihood of developing a substance use problem, and protective factors include beliefs and behaviors that decreases the probability of developing a substance use problem. Preliminary codes were identified by three researchers who first independently reviewed and coded transcripts and then met to determine consensus and revise codebook definitions as necessary [42]. We used triangulation, the method of employing several researchers to analyze the same participant data to reduce bias [43], with at least two of the three researchers reviewing each transcript and meeting to evaluate and discuss discrepancies and update codebooks. Previously coded transcripts were re-coded if codes were updated or added. Data coding was completed using NVivo qualitative data analysis software (QSR International Pty Ltd. v11, 2015) through an iterative process with themes and codes adapted as new information was collected and questions were added to interviews. Following coding, we used a thematic analysis [44] approach involving summarizing codes pertaining to our key findings, which are illustrated in the section below using illustrative, anonymized quotes. We used several methods to ensure trustworthiness of our descriptive qualitative study approach, including [45] expert review of our interview guide, codes, and key findings; iterative, team-based approach to codebook development and code application (ensure that all key domains of interest from our interview guide and the risk and protective strategy framework were captured in codes); and assessment of consistency in code application across research team members.

## Results

### Participant Characteristics and Overview of Findings

The sample included 20 participants (25% female, n* *=* *5) whose ages ranged from 21 to 70 years (median = 56.5 years; Table 1). Participants had a median AUDIT-C score of 6 (range 4–11) with 30% (n = 6) reporting current tobacco use and 15% (n = 3) current illicit substance use. From qualitative interviews, we identified three themes related to participants' beliefs and behaviors regarding opioid and alcohol use for pain management before and after surgery. We also categorized beliefs and behaviors into risk and protective factors within these themes. First, participants held varying positive and negative expectations about postoperative opioid use that were linked to their intentions to use opioids after surgery. Second, participants varied in their intentions to use alcohol as a pain management strategy before and after surgery. Third, participants came into surgery with pre-existing beliefs about alcohol and opioid co-use that appear to represent risk and protective factors. The variability in beliefs and intentions across these three themes suggest different clinical implications that likely require varying levels of intervention (see Table 2). There were no observed systematic differences between content or quality of phone vs in-person interviews.

**Table 1.** Participant and substance use characteristics

Variable	N = 20
Age	Median (56.5) Range (21–70)
Female	5
Race	
White	17
Black	2
Other	1
Hispanic ethnicity	1
AUDIT-C score	Median = 6 Range [4–11]
Current tobacco use (yes)	6
Current illicit drug use (yes)	3
Current prescription medication misuse (yes)	0

**Table 2:** Risk stratification of pre-operative patient factors and risk mitigation strategies for each theme

Theme	Risk Stratification	Patient Factors	Prevention and Intervention Strategy
Expectations and intentions to use opioids after surgery	Risk factors	Sees surgery as an opportunity to use a narcotic patient enjoys History of positive subjective effects of opioids such as euphoria and increased focus	Assess pain medication expectancies Motivational interviewing Alternative pain management Opioid monitoring Post-operative follow-up interventions
	Protective factors	Wants to avoid using opioids due to fear of addiction Past negative experiences using opioids (e.g. nausea)	Reinforce knowledge Provide accurate education about safe opioid use and risk mitigation
Use of alcohol for pain management	Risk factors	Past/current use of alcohol as a pain management strategy Prefers alcohol over opioids for pain management Curious about alcohol as a way to manage pain	Alcohol screening, brief intervention, and referral to treatment Referrals to pain clinics or specialists Opioid monitoring Alternative pain management education or referrals
	Protective factors	No history of using alcohol to manage pain No plans/desire to use alcohol to manage pain	Psychoeducation about alcohol and surgical health
Attitudes about alcohol and opioid co-use	Risk factors	Holds positive or permissive attitudes about mixing substances Current or past polysubstance use	Risk education Opioid monitoring Alternative pain management
	Protective factors	Has knowledge of risks associated with alcohol and opioid co-use No history or intentions of polysubstance use	Reinforce knowledge Provide accurate education about safe opioid use and drug interactions

#### Participant Expectations about Postoperative Opioid Use Were Linked to Intentions to Use Opioids after Surgery

##### Risk Factors

Some individuals held high-risk beliefs and intentions regarding using prescribed opioids for acute surgical pain management including positive anticipation of pain relief and psychological effects. For example, some participants described positive anticipation of opioid access, and saw surgery as an “opportunity” to use opioids, despite the risk of relapse:

*“I’m actually looking forward to some good pain pills. You know, after the surgery. If I’m in pain, I’ll make sure I get something. And if they give me some to take home, because I have pain and discomfort, I will take them. And I’ll take them as long as I have pain and discomfort. And I think I can manage it, you know. Because I don’t wanna be re-addicted to it.”*

Other participants looked forward to using opioids after surgery and reported positive expectations of opioids related to psychological effects:

“*Well, you're in pain and you take these oxy [oxycodone] and it’s like you sit there for 5 minutes or less and all of a sudden it just washes over you and you're in this place. Well, you're not wasted or anything. You just feel happier. You feel stupider, ya know…”*

Some participants report positive psychological and pain management experiences with opioids that led to dependence symptoms and having to slowly wean off:

*“There’s something about opioids that increased my level of focus….and boy I can get a lot done when I’m on those. When I’m on opioids, I feel they take the place of the alcohol but they have like a different…different reaction or a different feeling. The first time I had oxycodone, I hated it. But then I got used to them. And then you need more to get the same effect and what I do is I always cut myself back and I’m only taking half a tablet or whatever because I want them to last…. The last time I had a couple of refills and every time you get a refill they cut down the number that you get… and so they kind of wean you off it.”*

##### Protective Factors.

Not all participants held positive expectations about opioids’ effects. Some participants reported apprehension and fear regarding postoperative opioid use for acute pain management due to their addictive properties. Participants mentioned media coverage of the opioid epidemic and personal experience with friends and family becoming addicted to prescribed opioids as motivators to avoid using opioids:

*“I don’t want to get addicted to [opioids]. Just this year a friend of mine’s 22 or 23-year-old kid died in his sleep from an overdose of opioids.”*

Others cited their own insights into their personal vulnerability to addiction:

*“I believe I have an addictive personality to begin with and so that part makes me nervous, the addiction part. And also, just kind of being not myself and out of my mind. I don’t want to be out of my own mind or not have control over my own body.”*

Another subset of participants reported past negative side effects of opioid-based pain relievers that made them wary of this class of medications. These past negative experiences included nausea, difficulty with opioid dependence/tapering after past surgeries, and poorly managed pain. Some participants preferred not to use opioids if they could avoid them:

*“I got prescribed Percocet [oxycodone] the first time and it made me throw up and then I had been prescribed Vicodin [hydrocodone/acetaminophen] when I got my wisdom teeth taken out and … I did not like the way it made my head feel so, I just decided, I don’t need that stuff.”*

#### Participants Varied in Their Intentions to Use Alcohol for Pain Management

##### Risk Factors

Some individuals described using alcohol for pain management on a regular basis, even preferring it to opioids for pain management in some instances. Participants described alcohol as effective as an analgesic to manage persistent pain and aches:

*“And now I'm trying to get my hips fixed and my shoulder fixed, ‘cause that’s really bothering me, but then they put you through all this physical therapy and all this stuff, and you just, finally you just get fed up with it. And you suffer. Then when you start working, like mowing the lawn or doing the yard, cutting wood or whatever, then your whole body just starts hurting. And you just have a few drinks and loosen up a little bit and you're good to go. It helps you, you know?”*

In some instances, participants preferred alcohol over opioids for pain management:

*“Alcohol seems to help the most, you know, that seems to help more than the pain pills really…. cause you're sitting down not doing nothing too. I mean, you can't run around the block, mow the lawn.”*

One participant compared the physical experience of cold beer to “icing” his knee and emphasized the psychological experience of this pain management experience:

“*If it’s really, really throbbing, then I just…I get me one [beer]. Maybe it’s cuz it’s cold going through there and…I’m supposed to be icing it, so I…I grab me one out of the fridge and start drinking on that and it…it…it helps with the pain more mentally versus physically.*”

Some participants drank alcohol solely to manage psychological aspects of the pain experience, including to distract from or to take one’s mind off of pain:

*“It [alcohol] alters your brain, so when you have a drink, you're on a high, you’re buzzed. So you're not thinking about your pain. You're laughing. You're having fun….I’m not having a drink and crying. I’m having a drink and I’m having fun so I’m not thinking about my pain. I’m distracted from it, so I think that’s what it is. I don’t know that it’s taking the pain away, but I’m distracted…it’s a distraction from it.”*

##### Protective Factors

A few participants never used alcohol to manage pain. One reason cited was that they did not believe that alcohol would be effective in reducing or controlling any type of pain and may even exacerbate it:

*“I can't imagine alcohol ever taking away pain. I mean just like falling or running into something, and then just causing myself more discomfort.”*

Others had chronic pain but never used alcohol for pain management:

*“Definitely don’t use [alcohol] to cope with pain. I have chronic back prob-…lower back pain from herniated disks and the gel that’s not there anymore that’s supposed to be there, and so I have pain 24/7. When I roll over in the middle of the night, I wake up, and it takes me forever to get back to sleep, so I mean I have pain, but I definitely don’t use alcohol for it.”*

Others couldn’t imagine alcohol would ever be helpful for surgical pain but could be helpful for other types of pain:

*“Well, I don't think [alcohol] will help with pain…well, not with a big surgery, but with a surgery like that, but like something small, let's say, oh, I have a toothache.”*

Finally, there were other participants who had experimented with using alcohol for perioperative pain but found it did not help:

*“I had to stop taking ibuprofen as of yesterday and I was like well, wonder if the beer would help. I had 2 beers last night and it didn’t really help.”*

#### Participants Beliefs about Alcohol and Opioid Co-Use

##### Risk Factors.

Beliefs about concurrent use of alcohol and opioids varied. Some suggested ongoing risk of alcohol and opioid co-use. For example, some individuals lacked concern about alcohol and opioids co-use and actually thought the combination of alcohol opioids prolonged their effectiveness for pain management:

*“I had knee surgery. I had ACL replacement. I was playing rugby then, so I’d go to the rugby games on crutches, and I had my Vicodin and I’d have a beer or 2…. I think it makes it last longer. But I don’t think other than not following the directions… I didn't feel like I was abusing anything.”*

Some participants did not use alcohol and opioids together but explained that opioids served as a replacement for drinking alcohol, suggesting low risk of co-use but potential risks for new opioid problems:

*“I almost want to say the opioids are kind of are a replacement… to keep you from drinking. Ya know, cause your pain is relieved and your trauma’s not so bad.”*

Some participants did not want to mix alcohol and opioid use, and were therefore eager to finish their opioid-based prescription in order to “get back to drinking.” One contribution to this decision was viewing opioids as risker than alcohol in terms of addiction potential:

*“I…I wouldn’t be taking the Vicodin unless I needed it so then, I wouldn’t mess with the alcohol at all. My last surgery, I…I only took it like half a dose of Vicodin cuz I am more leery about pills and other things, narcotics and things like that and I’m more concerned about not abusing that in any way, more so than alcohol. Alcohol, I can be a little bit more relaxed with.”*

##### Protective Factors.

Protective beliefs for alcohol and opioid co-use included having no interest in or being wary of mixing alcohol and opioids. Some individuals stated that they were aware that they shouldn’t drink while taking medications and were absolute in their decision to not do so:

*“Almost every drug you take tells you not to take it with alcohol so when it comes to that kind of stuff, I pay attention to that.”*

## Discussion

This study explored elective surgical patients’ beliefs and behaviors related to using opioid-based pain relievers and alcohol to manage pain before and after surgery. Thematic analysis of in-depth qualitative interviews revealed three key findings. First, participants’ expectations about postoperative opioid use were linked to intentions to use opioids after surgery. Second, participants described great variations in how they employed or avoided alcohol for pain management. Third, participants held high and low-risk beliefs about alcohol and opioid co-use. Risk and protective factors were identified across these three themes suggesting the possibility of addiction risk stratification prior to surgery. With proper assessment, these risk factors could help identify patients most vulnerable to new opioid problems, at increased risk of unhealthy alcohol in the context of perioperative surgical pain, and/or at risk for potentially dangerous alcohol and opioid co-use following surgery. These findings have important implications for perioperative screening and intervention.

Participants had preexisting expectations about opioids’ effects based on past experiences and knowledge. These expectations were often linked to their intentions to use or avoid using opioids after surgery. This is consistent with literature on drug expectancies, or the degree to which individuals expect a substance to produce a variety of general and specific effects. Drug expectancies are strongly linked to actual drug use and the development of addiction [46]. Past research finds positive expectancies of opioid-based pain medication are predictive of opioid misuse and frequency of other drug use [47]. Our qualitative findings corroborate this research in the surgical context. These findings also suggest that assessing opioid-related expectancies could help predict opioid misuse after surgery [47]. We also found positive opioid-related expectancies were sometimes coupled with a lack of concern about risk of addiction or relapse. Research clearly links re-exposure to a drug as a strong trigger for craving and relapse [48]. Over-confidence in one’s ability to manage and stop using opioids coupled with positive expectancies is a potential red flag worth evaluating in future prevention research.

Our findings also highlight participants’ subjective experiences using alcohol to manage acute and chronic pain before and after surgery. Participants explained that alcohol helped them reduce pain leading up to surgery, and for some, it also helped relieve tension, reduce distraction, and enhance mood. This is consistent with research highlighting the potent analgesic effects of alcohol on experimentally-induced pain in laboratory settings [23] and extends this work by describing subjective experiences of alcohol in a ‘real-world’ sample of participants experiencing clinical pain prior to surgery. A subset of participants in this study reported a clear preference for alcohol relative to opioids for managing pain. This is a novel finding, worth further research investigation as it suggests surgical pain could increase alcohol use or accelerate the progression of alcohol use disorders in individuals who use alcohol to manage pain, particularly for those who transition from acute to persistent or chronic postoperative pain [49]. Conversely, some participants expressed protective beliefs and behaviors related to alcohol and pain management, including having no interest or desire to use alcohol for pain management. Future research including prospective trials should evaluate whether past experience and intentions for using alcohol as an analgesic is related to perioperative alcohol use and pain management.

Finally, concomitant alcohol and opioid use is a concern highlighted by our findings, particularly when individuals with alcohol problems are prescribed opioids for surgical pain. We found that some participants had lenient beliefs about co-use of alcohol and opioids. Other participants had knowledge that alcohol and opioids were risky and planned to avoid drinking alcohol while using opioid-based pain relievers. These qualitative findings add to the literature on alcohol and opioid co-use and overdose risk [50, 51]. Individuals who perceive or anticipate positive rather than negative consequences of alcohol and opioid of co-use may be at higher risk of actual co-use after surgery. Per the theory of planned behavior, beliefs about consequences are linked to behavioral intentions and subsequent behavior in the absence of other mitigating factors [52].

### Clinical Recommendations

The heterogeneity and spectrum of beliefs among individuals with unhealthy alcohol use undergoing elective surgery points to the need to first assess beliefs, then educate, and finally intervene strategically in this patient population (see Table 2). In terms of screening enhancement, pre-operative screening for unhealthy alcohol use, pain management intentions, and expectancies regarding opioid effects could be beneficial. Our findings also suggest intervention stratification depending on individuals’ risk level. Low-risk individuals could benefit from basic education about opioid use and addiction risks and reminders to avoid alcohol use while using prescribed opioids [53]. For individuals with specific risk factors, additional interventions are warranted, possibly including motivational interviewing [54], alternative pain management techniques (e.g., acupuncture, mindfulness-based treatments) [55, 56], and monitoring of opioid use with follow-up as appropriate. This should likely be done regardless of individuals’ confidence in their ability to control opioid use. An additional assessment of expectancies related to opioid-based pain medications [47] may further differentiate risk trajectories and identify those in need of more intensive interventions. Our findings suggest that individuals have clear preferences for and past experiences with opioids that could be elucidated through assessment and trigger more or less intensive prevention efforts in a stratified format.

Regarding alcohol use, improving alcohol screening prior to surgery could improve identification of individuals at risk. For those who report unhealthy alcohol use, additional interventions and referral to treatment may be warranted. Coordination with pain management specialists could benefit those who use alcohol to manage pain. Those with unhealthy alcohol use would also benefit from enhanced education and conversations about the risks of opioid and alcohol co-use including overdose and, in some cases, naloxone access when appropriate [57].

## Conclusion

Individuals with unhealthy alcohol use have unique pain management needs and addiction risks during the perioperative period. In this qualitative study, elective surgical patients described specific beliefs, behaviors, and intentions related to using and combining alcohol and opioids during the perioperative period. Risk and protective factors were identified across themes. Risk factors included positive expectancies of opioids’ pain relieving and euphoric effects, use of alcohol to manage pain, and perceiving positive, rather than negative, consequences of alcohol and opioid co-use. With proper assessment, these risk factors could help identify patients most vulnerable to new opioid problems and unhealthy alcohol use in the context of perioperative surgical pain.

## Notes

Funding sources: This work was supported by the National Institute for Alcohol Abuse and Alcoholism K23 AA023869. L.A.L. was supported by a Career Development Award (CDA 18-008) from the VA Health Services Research & Development Service. A.R.B. was supported by a K01 award from the National Institute of Drug Abuse (K01DA043412).
